# Focal Thyroid Incidentalomas on ^18^F-FDG PET/CT: A Systematic Review and Meta-Analysis on Prevalence, Risk of Malignancy and Inconclusive Fine Needle Aspiration

**DOI:** 10.3389/fendo.2021.723394

**Published:** 2021-10-20

**Authors:** J. F. de Leijer, M. J. H. Metman, A. van der Hoorn, A. H. Brouwers, S. Kruijff, B. M. van Hemel, T. P. Links, H. E. Westerlaan

**Affiliations:** ^1^ Department of Radiology, Medical Imaging Center, University Medical Center Groningen, University of Groningen, Groningen, Netherlands; ^2^ Department of Surgical Oncology, University Medical Center Groningen, University of Groningen, Groningen, Netherlands; ^3^ Department of Nuclear Medicine and Molecular Imaging, Medical Imaging Center, University Medical Center Groningen, University of Groningen, Groningen, Netherlands; ^4^ Department of Pathology, University Medical Center Groningen, University of Groningen, Groningen, Netherlands; ^5^ Department of Internal Medicine, Division of Endocrinology, University Medical Center Groningen, University of Groningen, Groningen, Netherlands

**Keywords:** thyroid, incidentaloma, FDG (^18^F-fluorodeoxyglucose)-PET/CT, thyroid nodule, thyroid cancer

## Abstract

**Background:**

The rising demand for ^18^F-fluorodeoxyglucose positron emission tomography with computed tomography (^18^F-FDG PET/CT) has led to an increase of thyroid incidentalomas. Current guidelines are restricted in giving options to tailor diagnostics and to suit the individual patient.

**Objectives:**

We aimed at exploring the extent of potential overdiagnostics by performing a systematic review and meta-analysis of the literature on the prevalence, the risk of malignancy (ROM) and the risk of inconclusive FNAC (ROIF) of focal thyroid incidentalomas (FTI) on ^18^F-FDG PET/CT.

**Data Sources:**

A literature search in MEDLINE, Embase and Web of Science was performed to identify relevant studies.

**Study Selection:**

Studies providing information on the prevalence and/or ROM of FTI on ^18^F-FDG PET/CT in patients with no prior history of thyroid disease were selected by two authors independently. Sixty-one studies met the inclusion criteria.

**Data Analysis:**

A random effects meta-analysis on prevalence, ROM and ROIF with 95% confidence intervals (CIs) was performed. Heterogeneity and publication bias were tested. Risk of bias was assessed using the quality assessment of diagnostic accuracy studies (QUADAS-2) tool.

**Data Synthesis:**

Fifty studies were suitable for prevalence analysis. In total, 12,943 FTI were identified in 640,616 patients. The pooled prevalence was 2.22% (95% CI = 1.90% - 2.54%, I^2^ = 99%). 5151 FTI had cyto- or histopathology results available. The pooled ROM was 30.8% (95% CI = 28.1% - 33.4%, I^2^ = 57%). 1308 (83%) of malignant nodules were papillary thyroid carcinoma (PTC). The pooled ROIF was 20.8% (95% CI = 13.7% - 27.9%, I^2^ = 92%).

**Limitations:**

The main limitations were the low to moderate methodological quality of the studies and the moderate to high heterogeneity of the results.

**Conclusion:**

FTI are a common finding on ^18^F-FDG PET/CTs. Nodules are malignant in approximately one third of the cases, with the majority being PTC. Cytology results are non-diagnostic or indeterminate in one fifth of FNACs. These findings reveal the potential risk of overdiagnostics of FTI and emphasize that the workup of FTI should be performed within the context of the patient’s disease and that guidelines should adopt this patient tailored approach.

## Introduction

^18^F-fluorodeoxyglucose (^18^F-FDG) positron emission tomography (PET) with computed tomography (CT) has become an important diagnostic tool in the assessment of malignancies and inflammatory diseases (1, 2). It is estimated that 2.2 million PET/CT scans were performed in the USA in 2019, with an estimated growth of 6% per year since 2013 (3). Due to this rise in imaging demand, incidentalomas are being discovered more often. Incidentalomas are incidentally found lesions unrelated to the clinical indication for ^18^F-FDG PET/CT (4). The incidence of ^18^F-FDG incidentalomas increases with age, which makes a further increase in incidence and financial impact likely due to population demographics change (5).

^18^F-FDG is a glucose analog that accumulates in metabolically active tissue like malignant tumors (6). Therefore, incidentalomas discovered on ^18^F-FDG PET/CT have a relatively high risk of malignancy (ROM) compared to incidentalomas detected by other imaging modalities (e.g. ultrasound). The overall prevalence of incidentalomas on whole body ^18^F-FDG PET/CT is 2.5% in patients with or without known or suspected cancer (4). Malignant lesions are most commonly found in the gastrointestinal tract, thyroid and lung (6).

Thyroid incidentalomas can be classified as either focal or diffuse. Diffuse ^18^F-FDG uptake in the thyroid is often caused by inflammatory disease, like (autoimmune) thyroiditis or Graves’ disease (7, 8). In contrast, focal ^18^F-FDG uptake is more likely caused by benign thyroid disease or malignancy, i.e. adenoma, thyroid carcinoma, metastasis of another origin or lymphoma. The most recent meta-analyses till 2014 showed focal thyroid incidentaloma (FTI) malignancy risks ranging from 34.6 to 37 percent (8–11).

Guidelines of the American Thyroid Association (ATA), American College of Radiology (ACR), European Thyroid Association (ETA) and British Thyroid Association (BTA) recommend ultrasound (US) guided fine needle aspiration cytology (FNAC) for patients with focal increased uptake in the thyroid gland as detected by ^18^F-FDG PET/CT (12–14). The guidelines are well-delineated and easy to adhere to, but seem to provoke a reflexive or habitual process that propel patients from incidental discovery of a thyroid nodule to FNAC and even surgery (15). Ultimately, this approach might contribute to a cascade effect of overdiagnostics and overtreatment, affecting the quality of life of these patients. Because the recommendations are strongly based on non-randomized retrospective studies, they are restricted in giving options and modifications to tailor diagnostics and to suit the individual patient with his or her specific characteristics and concerns.

Non-diagnostic or indeterminate results on cytopathology are assessed as undesirable yields of the diagnostic chain, resulting in repeat examinations and anxiety and uncertainty in patients. At the same time, doctors and patients seem to be indifferent or unaware of the impact of this potential hazard. Therefore, different from previous systematic reviews and meta-analyses, we looked beyond the prevalence and the ROM of FTI and also analyzed the risk of inconclusive FNAC (ROIF).

We aimed at exploring the extent of potential overdiagnostics by performing a systematic review and meta-analysis of the literature on the prevalence, ROM and ROIF of focal thyroid incidentalomas (FTI) on ^18^F-FDG PET/CT, thereby revealing opportunities to improve FTI management.

## Methods

### Literature Search

A systematic literature search was conducted using MEDLINE, Embase and Web of Science to identify relevant articles. Database keywords and text words were searched using thyroid neoplasms, PET and incidental findings including the subcategories and variants of these words as search terms. Similar terms were used for Embase and Web of Science (**Supplemental Table 1**). The search was restricted to articles published between January 2010 and June 2020, to provide an update to existing meta-analyses. Articles without an English abstract and conference abstracts were excluded. If insufficient data were reported, the authors were contacted to provide additional information. To expand our search, references of retrieved systematic reviews and meta-analyses were screened for additional studies.

**Figure 1:**
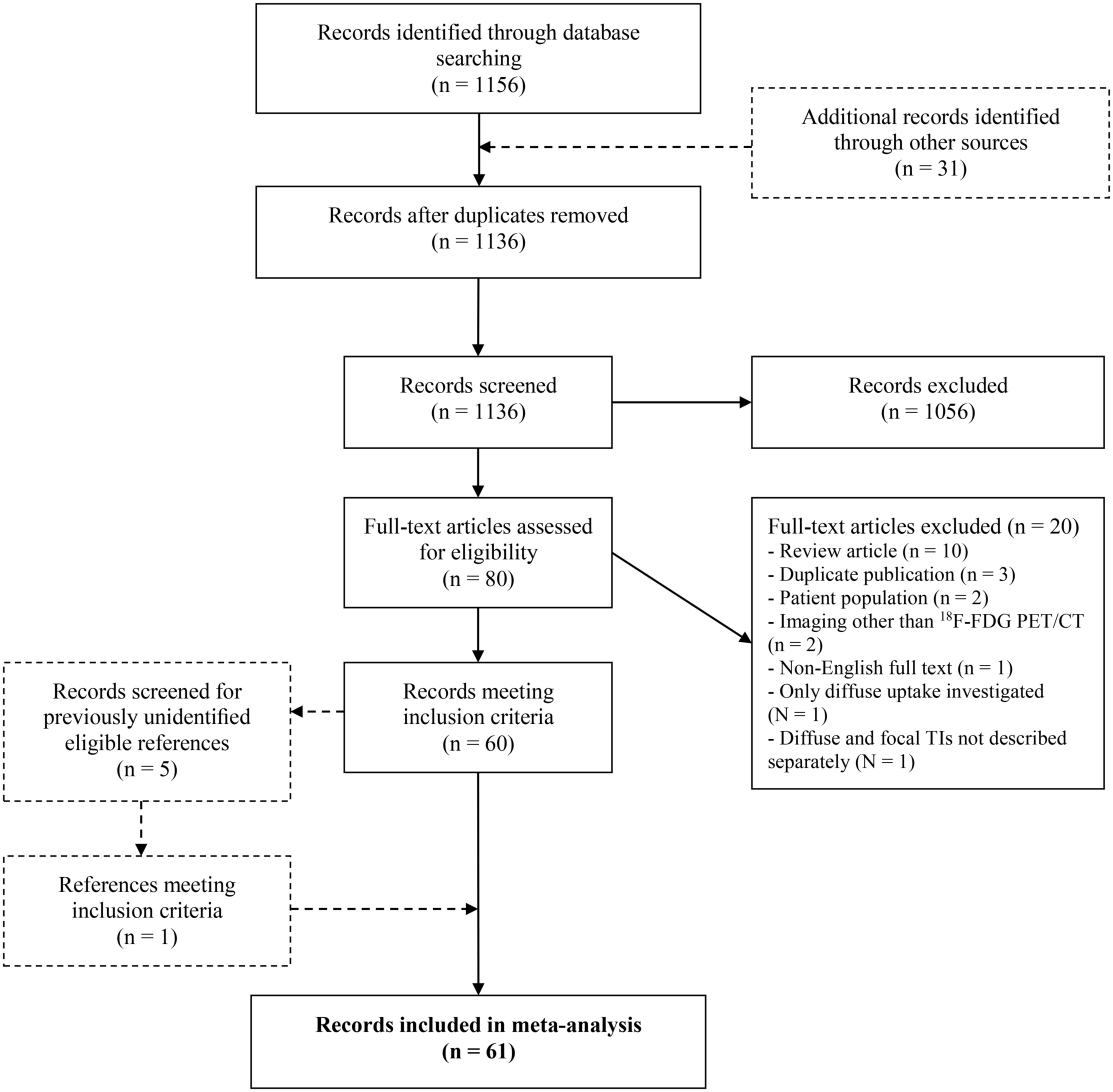
The Preferred Reporting Items for Systematic Reviews and Meta-Analyses (PRISMA) flow chart. ^18^F-FDG PET/CT, ^18^F-Fluorodeoxyglucose Positron Emission Tomography with Computed Tomography; FTI, Focal Thyroid Incidentaloma.

The complete search yielded 1156 articles and is displayed in accordance to the Preferred Reporting Items for Systematic Reviews and Meta-Analyses (PRISMA) in **Figure 1** (16).

### Study Selection

Retrospective and prospective cohort studies providing information on the prevalence of FTI on ^18^F-FDG PET/CT and/or ROM of ^18^F-FDG-avid FTI in patients with no prior history of thyroid disease were considered for inclusion. After duplicates were eliminated, studies were screened for eligibility based on title, abstract, and subsequently on full text by two authors (J.F.d.L., H.E.W.) independently. Disagreements on article inclusion were resolved by consensus reading by the same reviewers. Studies were excluded if: (a) only thyroid incidentalomas with diffuse uptake patterns were investigated or the results of focal and diffuse uptake patterns were not described separately. If both focal and diffuse thyroid incidentalomas were included in a study and described separately, the FTI were considered for further analysis. (b) they concerned a retrospective analysis of surgically treated FTI. (c) they concerned duplicate publications. If so, the study with the largest patient population was included. (d) the full article was written in a non-English language. Finally, 61 studies were included for analysis.

### Data Extraction

Data were extracted by three authors (J.F.d.L., M.J.H.M., H.E.W.) using a data extraction table. All full articles were analyzed by J.F.d.L. Duplicate data extraction was performed by either M.J.H.M. or H.E.W. Any discrepancies were resolved with consensus reading by a third reviewer (M.J.H.M. or H.E.W). The following data were collected for meta-analysis: the total number of ^18^F-FDG PET/CTs, the total number of FTI, the number of malignant and benign FTI and the number of FTI with a non-diagnostic or indeterminate cytopathology result after initial FNAC. Specific information regarding the pathological classification or description (based on either cytopathology and/or histopathology) of malignant FTI was collected as well. Hürthle cell and follicular carcinomas were considered as one group. Some studies had patients with multiple FTI. These FTI were considered as separate cases.

For analysis of ROM, FTI were classified according to cytopathology and histopathology. When both results were available, the histopathology result was used. The definition of a malignant cytopathology result was a description of “malignant” or “suspicious for malignancy” or, according to the Bethesda (B) classification, BV and BVI (17). Some studies used the British THY system as FNAC classification system. THY4 and BV were considered equally as well as THY5 and BVI, as described by the ETA (18). FTI with non-diagnostic/unsatisfactory (BI/THY1), atypia/follicular lesion of undetermined significance (AUS/FLUS) (BIII/THY3a) or (suspicious for) follicular neoplasm (BIV/THY3b) cytopathology were not defined as malignant or benign, unless histopathology or repeat cytopathology was done and decided otherwise (i.e. BII/BV/BVI and THY2/THY4/THY5). FTI were considered benign when they had benign cytopathology (BII/THY2) or histopathology. FTI classified according to ultrasound, scintigraphy or clinical follow-up were not considered for further analysis.

For analysis of ROIF, the number of FTI with initial BIII/indeterminate and BI/non-diagnostic cytopathology results were registered separately, independent from repeat FNAC or histopathology results used for ROM analysis.

### Quality Assessment

The quality of included studies was assessed using the quality assessment of diagnostic accuracy studies, QUADAS-2 (19). All studies were independently assessed by two reviewers (J.F.d.L., H.E.W.) and disagreements were resolved with consensus reading. QUADAS-2 divides the risk of bias of study methodology into four domains: patient selection, index test, reference test and flow and timing. Studies were considered to have a high risk of bias in the patient selection domain when a non-consecutive or non-random sample was used or inappropriate exclusion criteria were used like “patients with inconclusive cytology”. Studies were considered to have a high risk of bias in the index test domain when the ^18^F-FDG PET/CTs were interpreted or adjusted with knowledge of the FNAC results. Studies were only classified as high risk in the reference test domain when studies did not use the Bethesda classification to report FNAC results. The patient flow domain was classified as high risk when less than 50% of included patients received FNAC and/or surgery or FNAC was only performed after suspicious US. Domains were considered to be of unclear risk when insufficient information was given to assess methodological quality properly.

QUADAS-2 was used to assess applicability as well. In all studies the patient selection, index test and reference standard met the inclusion criteria and the question of the review.

### Statistical Analysis

Prevalence, ROM and ROIF of FTI were calculated using the data extracted from included studies. Regarding ROM, a proportion was calculated using the number of FTI investigated with FNAC and/or surgery as denominator and the number of FTI with malignant cytopathology or histopathology as nominator. ROIF was calculated using the number of initial FNAC as denominator and the number of non-diagnostic and indeterminate FNAC as nominator.

Data were pooled using a random effects model generated by the Cochrane Collaboration software, Review Manager (RevMan) 5.4 software. Heterogeneity was tested using the Chi-square test (p < 0.01) and Higgins and Thompson test to calculate the I^2^ statistic (20). As this demonstrated a heterogeneous study set, a random effects model was utilized to calculate pooled estimates. Publication bias was assessed using a funnel plot and weighed Egger’s regression test (21).

A forest plot was generated displaying the individual study prevalence, malignancy risk and percentage of indeterminate and non-diagnostic FNAC results with 95% Confidence Intervals (CIs) and the pooled estimates using the forestplot package in the R environment.

Subgroup analyses were done to identify sources of bias and heterogeneity in the data. Methodological quality and study characteristics (age, indication for PET, geography) were used to divide studies into subgroups. With regard to the latter, some parts of the world, i.e. South Korea, have a higher and faster increasing incidence of thyroid cancer, than other parts. Although this increase has mostly been attributed to overscreening and higher rates of diagnosis, a ‘true’ difference in incidence due to geographic variation in individual factors like obesity or genotype and environmental factors like iodine supplementation or radiation exposure also plays a role (22).

## Results

### Study Characteristics

Sixty-one studies were included in final analysis. The study characteristics are shown in **Table 1**. Most studies had a retrospective (n=55) study design. Thirty-eight of the studies included only patients who underwent ^18^F-FDG PET/CT for non-thyroidal oncological indications. The other studies also used ^18^F-FDG PET/CTs conducted for benign diseases and cancer screening in healthy subjects or did not specify the nature of the indication of the requested ^18^F-FDG PET/CT scans that were included. In total, 660,037 ^18^F-FDG PET/CTs were carried out and 13,603 FTI were identified. A brief overview of our data is shown in **Table 2**. Not all studies were suitable for both prevalence and malignancy risk analysis. Therefore, the data presented in the meta-analyses do not match the total number of ^18^F-FDG PET/CTs or the total number FTI.

**Table 1:** Study characteristics.

Name of author	Year	Country	Indication for PET	Study design	PETs	FTI (%)	% male	Age (SD)	Investigated FTI (FNAC/surgery)	Malignant FTI (%)
Kim et al. (23)	2010	South Korea	Cancer workup	RS	11623	159 (1.4)	13.8	62.5 (±10.7)	140 (140/0)	37 (26.4)
Kung et al. (24)	2010	Hong Kong	Cancer workup or cancer screening	RS	1407	45 (3.2)	26.7*	54.5 (±11.1)*	15 (4/11)	6 (40)
Zhai et al. (25)	2010	China	NS	RS	3580	115 (3.2)	44.3	49.6 (±10.3)	96 (76/20)	48 (50)
Czepczyński et al. (26)	2011	Poland	Cancer workup	RS	1925	71 (3.7)	NS	NS	20 (11/9)	9 (45)
Hsiao et al. (27)	2011	Taiwan	Malignant and benign diseases	RS	NS	199	34.2*	57.6 (±10.3)*	45 (29/16)	17 (37.8)
Kim et al. (28)	2011	South Korea	Cancer workup	RS	NS	50	20	58 (±10.6)	50 (48/2)	9 (18)
Nilsson et al. (29)	2011	Sweden	Cancer workup	RS	3641	37 (1)	29.7	64.6 (±9.1)	26 (15/11)	11 (42.3)
Nishimori et al. (30)	2011	Canada	Cancer workup	RS	4726	103 (2.2)	44,4**	56.8 (±13.2)**	39 (29/10)	9 (23.1)
Pagano et al. (31)	2011	Italy	Malignant and benign diseases	RS	11040	191 (1.7)	25*	64.1 (±12.5)*	36 (19/17)	14 (38.9)
Prichard et al. (32)	2011	Ireland	Cancer workup	RS	2105	35 (1.7)	25.7	64.4 (range 31-90)	20 (20/0)	8 (40)
Wong et al. (33)	2011	Australia	Cancer workup	RS	7896	188 (2.4)	34.6	65.3	59 (37/22)	17 (28.8)
Boeckmann et al. (34)	2012	USA	Cancer workup	RS	23384	690 (3)	36.9*	62.3 (±12)*	103 (4/99)	28 (27.2)
Bonabi et al. (35)	2012	Switzerland	Cancer workup	RS	3062	53 (1.7)	60**	67.5 (±8)**	42 (42/0)	10 (23.8)
Fujii et al. (36)	2012	Japan	Cancer workup	RS	NS	18	33.3	62.8 (±7.9)	18 (NS)	3 (16.7)
Kao et al. (37)	2012	Singapore	Cancer workup	RS	942	21 (2.2)	50**	71.3 (±5.4)**	6 (0/6)	3 (50)
Lee et al. (38)	2012	South Korea	Cervical cancer	RS	327	17 (5.2)	NS	56.3 (±10.7)	16 (13/3)	4 (25)
Pampaloni et al. (39)	2012	USA	Cancer workup	RS	8464	NS	25	59.3 (33-86)	32 (32/0)	13 (40.6)
Yang et al. (40)	2012	China	Cancer workup or cancer screening	RS	15948	281 (1.8)	34.2*	53.9 (±12.6)*	NS	NS
Bertagna et al. (41)	2013	Italy	Cancer workup	RS	49519	729 (1.5)	39.4	65.3	211 (139/72)	72 (34.1)
Kim et al. (42)	2013	South Korea	Cancer workup	RS	22674	483 (2.1)	16.1*	59 (±11.7)*	286 (285/1)	79 (27.6)
Kim et al. (43)	2013	South Korea	Cancer workup or cancer screening	RS	12119	262 (2.2)	25.6*	58.4 (±12.1)*	177 (140/37)	37 (20.9)
Achury et al. (44)	2014	Spain	Malignant and benign diseases	RS	4085	46 (1.1)	28.9	64 (26-85)	23 (23/0)	5 (21.7)
Brindle et al. (45)	2014	UK	Cancer workup	RS	7221	81 (1.1)	41.3	68	26 (26/0)	7 (26.9)
Choi et al. (46)	2014	South Korea	Cancer workup	RS	7914	171 (2.2)	36.4*	59.4 (±11.4)*	171 (103/68)	78 (45.6)
Elzein et al. (47)	2014	UK	Malignant and benign diseases	RS	1753	35 (2)	33.9	66 (26-84)	16 (10/6)	2 (12.5)
Marques et al. (48)	2014	Portugal	Cancer workup	RS	9374	60 (0.6)	18	62	23 (9/14)	14 (60.9)
Stangierski et al. (49)	2014	Poland	Malignant and benign diseases	RS	5520	122 (2.2)	32	60.7 (±12.1)	82 (60/22)	19 (23.2)
Yaylali et al. (50)	2014	Turkey	Cancer screening	RS	2000	57 (2.9)	40.4	60.9 (±14)	20 (20/0)	7 (35)
Adas et al. (51)	2015	Turkey	Cancer workup	RS	2654	25 (0.9)	26.5	57.7	25 (16/9)	11 (44)
Agrawal et al. (52)	2015	UK	Malignant and benign diseases	RS	29300	147 (0.5)	31.9*	63.2 (±14)*	41 (31/10)	9 (22)
Chun et al. (53)	2015	South Korea	Cancer workup or cancer screening	RS	2584	52 (2)	33.3*	63.4 (± 10.9)*	36 (18/18)	15 (41.7)
Gavriel et al. (54)	2015	Australia	Cancer workup	RS	1034	51 (4.9)	39.2	60 (range 25-81)	48 (32/16)	21 (43.8)
Jamsek et al. (55)	2015	Slovenia	Cancer workup	RS	5911	148 (2.5)	35.1	64.5 (±11.8)	52 (34/18)	12 (23.1)
Kim et al. (56)	2015	South Korea	Cancer workup or cancer screening	RS	23462	493 (2.1)	21.5*	51.2 (±10.7)*	200 (128/72)	49 (24.5)
Sharma et al. (57)	2015	UK	Cancer workup	RS	235	9 (3.8)	33.3	57 (range 42-74)	9 (1/8)	6 (66.7)
Yoon et al. (58)	2015	South Korea	Cancer workup	RS	NS	116	44.8*	60 (±12.6)*	87 (60/27)	40 (46)
Barrio et al. (59)	2016	USA	Cancer workup	RS	6216	243 (3.9)	32.5	68 (41-88)	67 (67/0)	21 (31.3)
Demir et al. (60)	2016	Turkey	Cancer workup	RS	1450	39 (2.7)	59.6	58.5 (±10.6)	32 (32/0)	10 (31.3)
Flukes et al. (61)	2016	Australia	Cancer workup	RS	27851	154 (0.6)	55.8	65.9 (range 35-92)	53 (36/17)	21 (39.6)
Hassan et al. (62)	2016	Pakistan	Cancer workup	RS	10012	93 (0.9)	39.9	53 (20-78)	50 (50/0)	24 (48)
Şencan Eren et al. (63)	2016	Turkey	Cancer workup	PS	4204	NS	32.8	60 (±12.6)	49 (37/12)	20 (40.8)
Vaish et al. (64)	2016	India	Cancer workup	RS	37000	61 (0.2)	21.3	54.5 (±11.9)	26 (23/3)	7 (26.9)
Hagenimana et al. (65)	2017	Canada	NS	RS	40914	304 (0.7)	38.3*	61.9 (±11.2)*	161 (115/46)	NS
Li et al. (66)	2017	USA	Cancer workup	RS	NS	20	40	68 (41-88)	20 (9/11)	10 (50)
Makis et al. (67)	2017	Canada	Cancer workup	RS	7252	157 (2.2)	24.2*	62.2 (±13.7)*	57 (0/57)	14 (24.6)
Ozderya et al. (68)	2017	Turkey	Cancer workup	RS	6873	135 (2.0)	32.9*	62 (±11)*	76 (50/26)	35 (46.1)
Pak et al. (69)	2017	South Korea	Cancer workup or cancer screening	RS	28824	332 (1.2)	31.1*	60.7*	238 (238/0)	62 (26.1)
Sollini et al. (70)	2017	Italy	Malignant and benign diseases	RS	17104	453 (2.6)	36.4*	62 (±15)*	55 (33/22)	18 (32.7)
Thuillier et al. (71)	2017	France	Malignant and benign diseases	PS	10118	131 (1.3)	38.9*	64.2 (±11.6)*	62 (43/19)	10 (16.1)
Chung et al. (72)	2018	South Korea	Malignant and benign diseases	RS	96942	4672 (4.8)	24.5*	NS	1342 (1342/0)	496 (37)
Pattison et al. (73)	2018	Australia	Cancer workup	RS	45680	500 (1.1)	35.1*	66 (16-96)*	131 (95/36)	47 (35.9)
Sager et al. (74)	2018	Turkey	Cancer workup	RS	12796	221 (1.7)	24.9	NS	126 (126/0)	43 (34.1)
Abdel-Halim et al. (75)	2019	Denmark	Malignant and benign diseases	PS	NS	104	31.7	67 (range 34-90)	104 (53/51)	23 (22.1)
Kumar et al. (76)	2019	India	Malignant and benign diseases	PS	1016	23 (2.3)	25.9	NS	19 (16/3)	5 (26.3)
Larg et al. (77)	2019	Romania	Cancer workup	PS	6900	126 (1.8)	27	62 (±13)	29 (20/9)	7 (24.1)
Lin et al. (78)	2019	Taiwan	Cancer workup	RS	NS	74	43.2	60.1	70 (70/0)	19 (27.1)
Oven et al. (79)	2019	Turkey	Cancer workup	RS	1840	40 (2.2)	35	58 (range 36-84)	40 (40/0)	14 (35)
Shi et al. (80)	2019	China	Malignant and benign diseases	RS	6753	NS	31	49.5 (±13.7)	87 (0/87)	52 (59.8)
Bakhshayesh Karam et al. (81)	2020	Iran	Cancer workup	PS	1126	78 (6.9)	50*	51 (22-76)*	18 (18/0)	3 (16.7)
Kamakshi et al. (82)	2020	India	Malignant and benign diseases	RS	1737	204 (11.7)	30.9	51	29 (29/0)	3 (10.3)
Trimboli et al. (83)	2020	Switzerland	Malignant and benign diseases	RS	NS	79	39.2	68	75 (NS)	21 (28)

NS, Not Specified; PET, Positron Emission Tomography; FTI, Focal Thyroid Incidentaloma; FNAC, Fine Needle Aspiration Cytology; RS, Retrospective; PS, Prospective.

*Characteristics of patients investigated with FNAC or surgery.

**Characteristics of patients with a malignant FTI.

**Table 2:** Summary table.

	Number (% of FTI, % of FTI with pathological description*)
18F-FDG PET/CT	660,037
FTI	13,603
Cyto- or histopathology available	5151 (37.9%)
Malignant FTI	1714 (12.6%)
FTI with pathological description*	1584 (11.6%)
Papillary thyroid cancer	1308 (9.6%, 82.6%)
Follicular thyroid cancer	111 (0.8%, 7%)
Medullary thyroid cancer	34 (0.3%, 2.2%)
Anaplastic thyroid cancer	9 (0.1%, 0.7%)
Metastasis	97 (0.7%, 6.1%)
Lymphoma	13 (0.1%, 0.8%)
Other	12 (0.1%, 0.8%)

^18^F-FDG PET/CT, ^18^F-Fluorodeoxyglucose Positron Emission Tomography with Computed Tomography; FTI, Focal Thyroid Incidentaloma. *Based on either cytopathology or histopathology. The remaining nodules were described as “malignant”, but not specified.

### Quality Assessment

The methodological quality of the included studies is summarized in **Supplemental Table 2**.

In the patient selection domain, 17 studies were considered as high risk due to inappropriate exclusion criteria (27, 39, 42, 46, 54, 56, 58, 59, 62, 63, 67, 69, 70, 72, 76, 80, 83). We considered another 6 studies to be of high risk because a non-consecutive sample of patients was enrolled (24, 28, 29, 31, 66, 79). One study included only patients with a history of cervical cancer and was therefore classified as high risk (38).

In the index test domain, 16 studies were classified as being of unclear risk of bias as it was not described how ^18^F-FDG PET/CTs were assessed (24, 30, 32, 35, 39, 45, 47, 51, 52, 61, 65, 66, 73–75, 79).

Thirty-six studies did not use the Bethesda classification to report FNAC results and were therefore classified as high risk in the reference test domain (23–29, 31–37, 39–41, 44, 45, 47, 48, 50–54, 57, 59, 60, 67, 70, 74, 79–81, 83).

In the patient flow domain, 27 studies were classified as high risk because not all included patients received FNAC and/or surgery (24, 26, 27, 30, 31, 33, 34, 37, 39, 41, 45, 47, 48, 50, 52, 55, 56, 59, 61, 63, 64, 67, 70, 71, 73, 81, 82). Another 8 studies were considered to have a high risk of bias because FNACs were performed only after suspicious US (25, 40, 44, 51, 62, 66, 72, 77).

### Publication Bias

**Figure 2:**
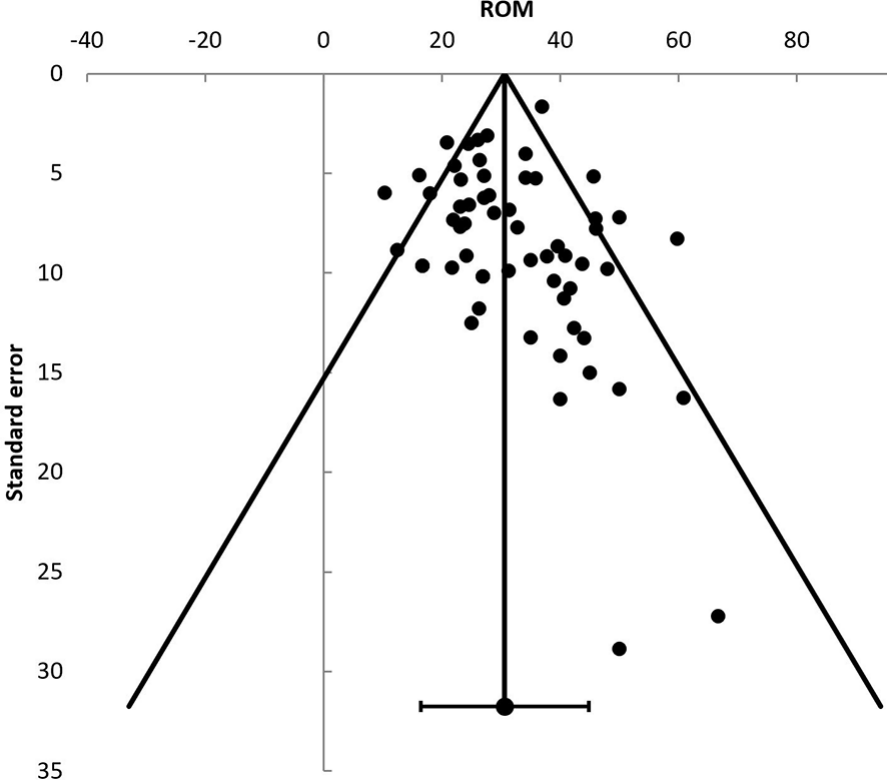
Funnel plot displaying individual studies risk of malignancy (ROM). ROM, risk of malignancy.

Publication bias was assessed using the malignancy risks reported in the included studies (**Figure 2**). An Egger’s regression showed no significant (t = 0.65, p = 0.52) funnel plot asymmetry.

### Prevalence

Fifty studies provided information regarding the prevalence of ^18^F-FDG-avid FTI. Of the 11 excluded studies, in 3 the prevalence data were not provided separately for focal and diffuse thyroid incidentalomas (39, 63, 80) and 8 did not report on the total number of ^18^F-FDG PET/CTs (27, 28, 36, 58, 66, 75, 78, 83).

A total of 640,616 patients undergoing ^18^F-FDG PET/CT were described. In this population, 12,943 FTI were identified. FTI prevalence ranged between studies, from 0.16% to 11.74%. The pooled prevalence of ^18^F-FDG-avid FTI was 2.22% (95% CI = 1.90% - 2.54%, I^2^ = 99%) (**Table 3**).

**Table 3:** Prevalence of FTI (random effects, I^2^ = 99%, symbol size reflects weight).

Study	PETs	FTI	Prevalence [95% CI]	
Vaish et al. (64)	37000	61	0.17 [0.13, 0.21]	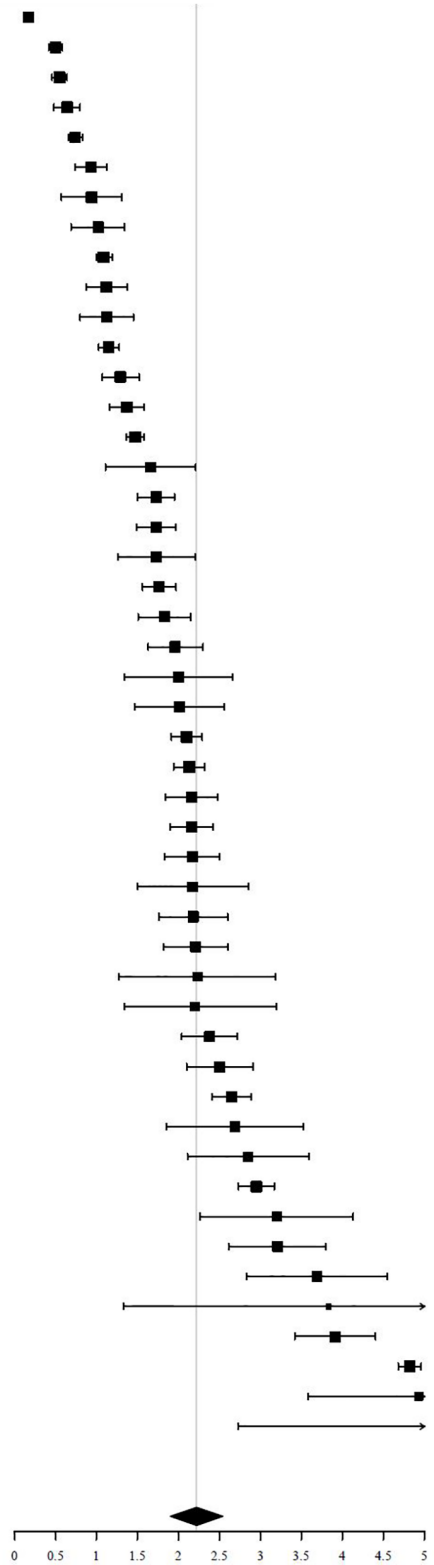
Agrawal et al. (52)	29300	147	0.50 [0.42, 0.58]	
Flukes et al.(61)	27851	154	0.55 [0.45, 0.65]	
Marques et al. (48)	9374	60	0.64 [0.48, 0.80]	
Hagenimana et al. (65)	40914	304	0.74 [0.66, 0.82]	
Hassan et al. (62)	10012	93	0.93 [0.73, 1.13]	
Adas et al. (51)	2654	25	0.94 [0.57, 1.31]	
Nilsson et al. (29)	3641	37	1.02 [0.69, 1.35]	
Pattison et al. (73)	45680	500	1.10 [1.00, 1.20]	
Brindle et al. (45)	7221	81	1.12 [0.87, 1.37]	
Achury et al. (44)	4085	46	1.13 [0.80, 1.46]	
Pak et al. (69)	28824	332	1.15 [1.03, 1.27]	
Thuillier et al. (71)	10118	131	1.30 [1.08, 1.52]	
Kim et al. (23)	1623	159	1.37 [1.15, 1.59]	
Bertagna et al. (41)	49519	729	1.47 [1.35, 1.59]	
Prichard et al. (32)	2105	35	1.66 [1.11, 2.21]	
Sager et al. (74)	12796	221	1.73 [1.49, 1.97]	
Pagano et al. (31)	1040	191	1.73 [1.48, 1.98]	
Bonabi et al. (35)	3062	53	1.73 [1.26, 2.20]	
Yang et al. (40)	15948	281	1.76 [1.54, 1.98]	
Larg et al. (77)	6900	126	1.83 [1.52, 2.14]	
Ozderya et al. (68)	6873	135	1.96 [1.63, 2.29]	
Elzein et al. (47)	1753	35	2.00 [1.33, 2.67]	
Chun et al. (53)	2584	52	2.01 [1.46, 2.56]	
Kim et al. (56)	23462	493	2.10 [1.90, 2.30]	
Kim et al. (42)	22674	483	2.13 [1.93, 2.33]	
Choi et al. (46)	7914	171	2.16 [1.83, 2.49]	
Kim et al. (42)	12119	262	2.16 [1.91, 2.41]	
Makis et al. (67)	7252	157	2.17 [1.84, 2.50]	
Oven et al. (79)	1840	40	2.17 [1.50, 2.84]	
Nishimori et al. (30)	4726	103	2.18 [1.75, 2.61]	
Stangierski et al. (49)	5520	122	2.21 [1.82, 2.60]	
Kao et al. (37)	942	21	2.23 [1.27, 3.19]	
Kumar et al. (76)	1016	23	2.26 [1.34, 3.18]	
Wong et al. (33)	7896	188	2.38 [2.05, 2.71]	
Jamsek et al. (55)	5911	148	2.50 [2.09, 2.91]	
Sollini et al. (70)	17104	453	2.65 [2.41, 2.89]	
Demir et al. (60)	1450	39	2.69 [1.85, 3.53]	
Yaylai et al. (50)	2000	57	2.85 [2.11, 3.59]	
Boeckmann et al. (34)	23384	690	2.95 [2.73, 3.17]	
Kung et al. (24)	1407	45	3.20 [2.26, 4.14]	
Zhai et al. (25)	3580	115	3.21 [2.62, 3.80]	
Czepcyński et al. (26)	1925	71	3.69 [2.83, 4.55]	
Sharma et al. (57)	235	9	3.83 [1.32, 6.34]	
Bariio et al. (59)	6216	243	3.91 [3.42, 4.40]	
Chung et al. (72)	96942	4672	4.82 [4.68, 4.96]	
Gavriel et al. (54)	1034	51	4.93 [3.58, 6.28]	
Lee et al. (38)	327	17	5.20 [2.73, 7.67]	
Bakhshayesh Karam et al. (81)	1126	78	6.93 [5.40, 8.46]	
Kamakshi et al. (82)	1737	204	11.74 [10.13, 13.35]	
**Summary**			**2.22 [1.90, 2.54)**	

PET, Positron Emission Tomography; FTI, Focal Thyroid Incidentaloma.

Subgroup analysis on age showed that prevalence of FTI was significantly lower in studies with a mean age > 60 (N = 27) than in studies with a mean age < 60 (N = 16). The pooled proportion in the > 60 subgroup was 1.76% (95% CI = 1.50% - 2.01%, I^2^ = 98%) and in the < 60 subgroup 2.91% (95% CI = 2.24% - 3.58%, I^2^ = 98%).

Subgroup analysis based on the ^18^F-FDG PET/CT indication showed no difference between studies including exclusively patients undergoing ^18^F-FDG PET/CT for non-thyroidal oncological indications (N = 31, prevalence 2.07%, 95% CI = 1.73% - 2.40%), compared to studies also including ^18^F-FDG PET/CTs conducted for benign diseases and cancer screening (N = 19, prevalence, 2.44%, 95% CI = 1.80% - 3.07).

Subgroup analysis based on geographic location showed that studies carried out in South Korea (N = 9, prevalence 2.45%, 95% CI = 1.10% - 3.79%) did not have a significantly different pooled prevalence than studies carried out in other countries (N = 41, prevalence 2.09%, 95% CI = 1.83% - 2.35%).

Finally, subgroup analysis using studies that were classified as “low risk” in the patient selection domain (studies with a consecutive design and appropriate exclusion criteria) (N = 34) *versus* studies that were classified as “high risk” (N = 16) did not result in significantly different pooled prevalences. The prevalence in the “low risk” subgroup was 1.98% (1.70% - 2.25%) and the prevalence was 2.57% (1.80% - 3.33%) in the “high risk” subgroup.

### Malignancy Risk

A total of 5151 FTI in 59 studies had cyto- or histopathology results available. One of two excluded studies did not provide sufficient information to calculate the ROM (65), the other did not adequately specify their eligibility criteria for further characterization with FNAC and only performed FNAC in a small part of their included patiënts (40).

Of the 5151 included FTI, 1714 FTI were malignant. The pooled ROM was 30.8% (95% CI = 28.1% - 33.4%, I^2^ = 57%) (**Table 4**). Of the 1714 malignant nodules, 1584 had a final pathological description available (based on either cytopathology or histopathology). The remaining 130 nodules were described as “malignant”, but not specified. Of these 1584 FTI with a pathological description available, 1462 (92%) were of thyroidal origin and 1308 (83%) were papillary thyroid cancer (PTC).

**Table 4:** Risk of malignancy of FTI (random effects, I^2^ = 57%, symbol size reflects weight).

Study	FTI	Malignant	ROM [95% CI	
Kamakshi et al. (82)	29	3	10.34 [-1.36,22.04]	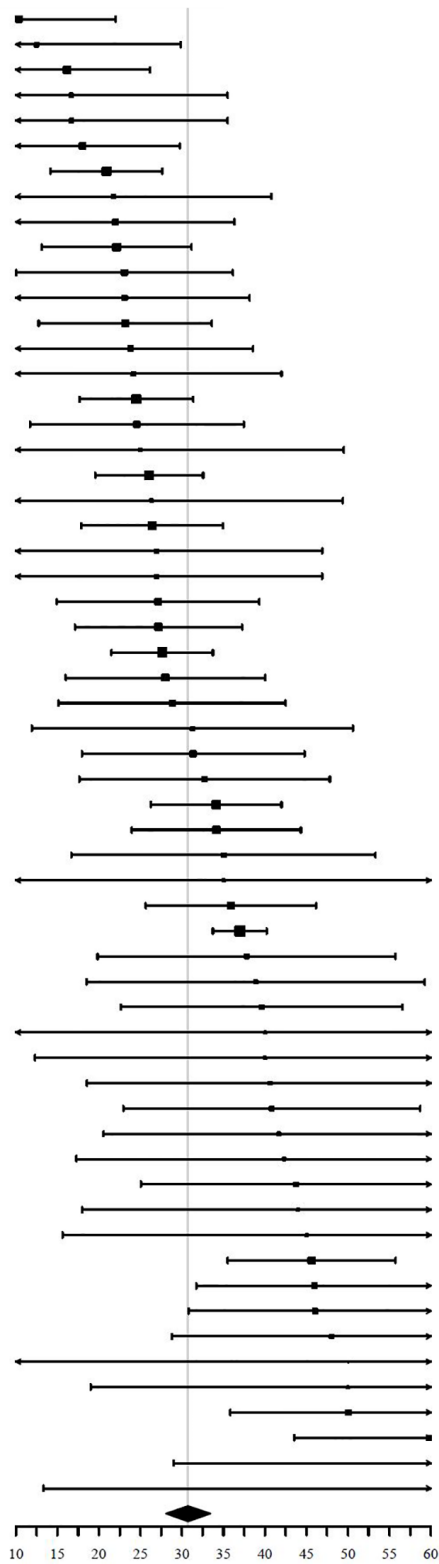
Elzein et al. (47)	16	2	12.50 [-4.83,29.83]	
Thuillier et al. (71)	62	10	16.1 [6.13,26.13]	
Bakhshayesh Karam et al. (81)	18	3	16.67 [-2.18,35.52]	
Fujii et al. (36)	18	3	16.67 [-2.18,35.52]	
Kim et al. (28)	18	9	18..00 [6.24,29.76]	
Kim et al. (42)	177	37	20.904.16,27.64]	
Achury et al. (44)	23	5	21.74 [2.69,40.79]	
Agrawal et al. (52)	41	9	21.95 [7.60,36.30]	
Abdel-Halim et al. (75)	104	23	22.12 [3.08,31.16]	
Jamsek et al. (55)	52	12	23.08 [0.01,36.13]	
Nishimori et al. (30)	39	9	23.08 [8.01, 38.15]	
Stangierski et al. (49)	82	19	23.17 [2.74, 33.60]	
Bonabi et al. (35)	42	10	23.81 [9.05, 38.57]	
Larg et al. (77)	29	7	24.14 [6.27, 42.01]	
Kim et al. (56)	200	49	24.50 [7.64, 31.36]	
Makis et al. (67)	57	14	24.56 [11.70, 37.42]	
Lee et al. (38)	16	4	25.0 [0.50, 49.50]	
Pak et al. (69)	238	62	26.05 [19.56, 32.54]	
Kumar et al. (76)	19	5	26.3 [3-25, 49.39]	
Kim et al. (23)	140	37	26.43 [17.92, 34.94]	
Brindle et al. (45)	26	7	26.9 [6.97, 46.87]	
Vaish et al. (64)	26	7	26.92 [6.97, 46.87]	
Lin et al. (78)	70	19	27.14 [14.93, 39.35]	
Boeckmann et al. (34)	103	28	27.18 [17.11, 37.25]	
Kim et al. (42)	286	79	27.62 [21.52, 33.72]	
Trimboli et al. (83)	75	21	28.00 [16.02, 39.98]	
Wong et al. (33)	59	17	28.81 [15.11, 42.51]	
Demir et al. (60)	32	10	31.25 [11.89, 50.61]	
Barrrio et al. (59)	67	21	31.34 [17.93, 44.75]	
Sollini et al. (70)	55	18	32.73 [17.62, 47.84]	
Bertagna et al. (41)	211	72	34.12 [26.24, 42.00]	
Sager et al. (74)	126	43	34.13 [23.94, 44.32]	
Oven et al. (79)	40	14	35.00 [16.67, 53.33]	
Yaylali et al. (50)	20	7	35.00 [9.07, 60.93]	
Pattison et al. (73)	131	47	35.88 [15.63, 46.13]	
Chung et al. (72)	1342	496	36.96 [33.71, 40.21]	
Hsiao et al. (27)	45	17	37.78 [19.83, 55.73]	
Pagano et al. (31)	36	14	38.89 [18.53, 59.25]	
Flukes et al. (61)	53	21	39.62 [22.67, 56.57]	
Kung et al. (24)	15	6	40.00 [7-99, 72.01]	
Prichard et al. (32)	20	8	40 [12.29, 67.71]	
Pampaloni (39)	32	13	40.63 [18.54, 62.72]	
Sencan Eren et al. (63)	49	20	40.82 [22.93, 58.71]	
Chun et al. (53)	36	15	41.67 [20.58, 62.76]	
Nilsson et al. (29)	26	11	42.31 [17.30, 67.32]	
Gavriel et al. (54)	48	21	43.75 [25.03, 62.47]	
Adas et al. (51)	25	11	44.00 [17.99, 70.01]	
Czepcyński et al. (26)	20	9	45.00 [15.60, 74.40]	
Choi et al. (46)	171	78	45.61 [35.50, 55.72]	
Yoon et al. (58)	87	40	45.98 [31.73, 60.23]	
Ozderya et al. (68)	76	35	46.05 [30.80, 61.30]	
Hassan et al. (62)	50	24	48.00 [28.79, 67.21]	
Kao et al. (37)	6	3	50.00 [-6.58, 106.58]	
Li et al. (66)	20	10	50.00 [19.01, 80.99]	
Zhai et al. (25)	96	48	50.00 [35.85, 64.15]	
Shi et al. (80)	87	52	59.77 [43.52, 76.02]	
Marques et al. (48)	23	14	60.87 [28.98, 92.76]	
Sharma et al. (57)	9	6	66.67 [13.32, 120.02]	
**Summary**		**30.75**	**28.06, 33.43**	

ROM, Risk of Malignancy; FTI, Focal Thyroid Incidentaloma.

A significant difference in pooled ROM between age subgroups was not found. Studies with a mean age > 60 years (N = 33) showed a ROM of 30.5% (95% CI = 27.6% - 33.4%), similar to the ROM of 31.8% (95% CI = 25.8% - 37.7%) in studies with a mean age < 60 years (N = 19). The ROM was not significantly different between studies including only patients undergoing ^18^F-FDG PET/CT for non-thyroidal oncological indications (N = 38) and studies including also ^18^F-FDG PET/CTs with non-oncological indications and for cancer screening (N = 21). The ROM was 32.2% (95% CI = 29.2% - 35.1%, I^2^ = 31%) in the oncology subgroup and 28.5% (95% CI = 23.6% - 33.4%, I^2^ = 75%) in the subgroup with other indications.

Finally, a subgroup analysis based on QUADAS-2 was performed. Patient selection, reference test and flow and timing were tested independently with “low risk” and “high risk” as subgroups. No significant difference in pooled ROM could be demonstrated.

### Inconclusive Cytopathology

Fifteen studies could be used to investigate the pooled ROIF after initial FNAC. Reasons for exclusion were: (1) missing data of non-diagnostic and indeterminate FNAC results (N = 28) (23–25, 27–29, 32, 33, 35–37, 39, 40, 51, 53, 54, 59, 60, 62, 64, 65, 67, 69, 74, 77, 80, 81, 83). (2) inconsequent description of data of non-diagnostic or indeterminate FNAC results (N = 5) (41, 45, 52, 66, 73). (3) missing separate data of BIII and BIV or THY3a and THY3b categories (N = 6) (31, 47, 57, 70, 75, 76). (4) description of either non-diagnostic or indeterminate FNAC results and not both subgroups (N = 7) (34, 38, 46, 48, 50, 78, 79).

Two of the 15 included studies did not use the Bethesda classification to report FNAC results (26, 44). They reported inconclusive results as either “non-diagnostic” or “indeterminate”.

In total, 2590 nodules were included for analysis of ROIF. Of these, 734 had a non-diagnostic or indeterminate FNAC result (219 non-diagnostic, 515 indeterminate). The pooled proportion of inconclusive FNAC results was 20.8% (95% CI = 13.7% - 27.9%, I^2^ = 92%) (**Table 5**).

**Table 5:** Risk of inconclusive FNAC of FTI (random effects, I^2^ = 92%, symbol size reflects weight).

Study	FNAC	Inconclusive	ROIF [95% CI]	
Yoon et al. (58)	87	6	6.90 [1.37, 12.43]	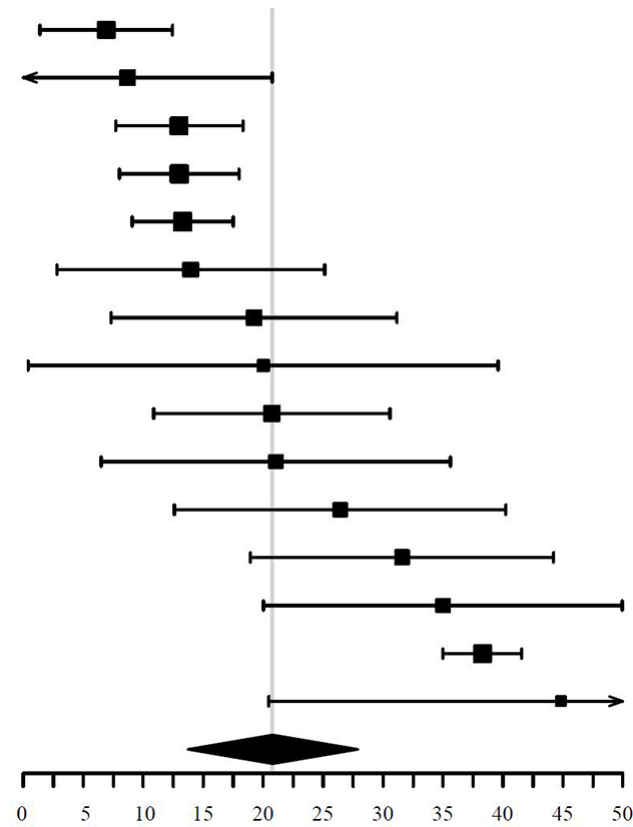
Achury et al. (44)	23	2	8.70 [-3.35, 20.75]	
Kim et al. (42)	177	23	1 2.99 [7.68, 1 8.30]	
Kim et al. (56)	200	26	13.00 [8.00, 18.00]	
Kim et al. (42)	286	38	1 3.29 [9.06, 1 7.52]	
Sencan Eren et al. (63)	43	6	13.95 [2.78, 25.1 2]	
Jamsek et al. (55)	52	10	19.23 [7.31, 31.1 5]	
Czepczyński et al. (26)	20	4	20.00 [0.40, 39.60]	
Stangierski et al. (49)	82	17	20.73 [10.87, 30.59]	
Nishimori et al. (30)	38	8	21.05 [6.47, 35.63]	
Flukes et al. (61)	53	14	26.42 [12.58, 40.26]	
Ozderya et al. (68)	76	24	31 .58 [18.94, 44.22]	
Thuillier et al. (71)	60	21	35.00 [20.03, 49.97]	
Chung et al. (72)	1364	522	38.27 [34.98, 41.56]	
Kamakshi et al. (82)	29	13	44.83 [20.47, 69.19]	
**Summary**			**20.81 [13.70, 27.92]**	

FNAC, Fine Needle Aspiration Cytology; ROIF, Risk of Inconclusive FNAC.

## Discussion

The present systematic review and meta-analysis shows a pooled prevalence of ^18^F-FDG-avid focal thyroid incidentalomas (FTI) of 2.2%. Malignancy is found in about one third of the FTI, the vast majority being papillary thyroid cancer (PTC). Non-diagnostic or indeterminate FNAC results are seen in approximately 21% of FNACs, meaning diagnostic uncertainty and new decision making.

This study can be considered as an update with inclusion of studies published in the last 10 years, using newer generations of PET/CT scanners. A major distinction from previous reviews is that we analyzed the risk of inconclusive FNAC (ROIF) with the purpose of estimating the encountered difficulties of the diagnostic chain. Both the risk of malignancy (ROM) and ROIF are key findings of our analyses and illustrate the necessity of tailoring the diagnostics of FTI to suit the preferences and context of the individual patient. The found ROIF (21%) is comparable to the ROIF found in a general population with thyroid nodules (23%) (84). Our findings concerning prevalence and ROM of FTI on ^18^F-FDG-PET/CT are similar to those in previous meta-analyses, which found FTI prevalences varying between 1.6% and 2.5% and ROM of 35-37% (8–11).

The substantial ROM along with the common finding and still rising number of FTI on ^18^F-FDG PET/CT seem to justify further diagnostics. However, the general approach to continue to ultrasound guided FNAC might contribute to overdiagnostics and overtreatment of benign nodules and (small) PTC. Similarly, the accompanying undiagnostic or indeterminate findings on cytopathology might require repeat FNACs or surgeries in one-fifth of patients. Additional undesirable consequences of this straightforward approach might be anxiety and interferences with definitive treatment planning, in particular in patients with other malignancies making up the main indication for ^18^F-FDG-PET/CT. Moreover, the impact of diagnosing a thyroid malignancy on overall survival in patients with other malignancies is questionable, not to mention the significant health care costs of incidentally detected findings (5, 73, 85, 86). Finally, the general recommendation ignores the importance of engaging patients in making decisions.

Given these points, the options of “inaction” or alternative action and active investigation according to current guidelines need to be explored evenly and the preferred option should be consistent with the patients’ wishes and preferences. The clinical context needs to be weighed carefully on the possible scenarios after FNAC and the clinical impact of an incidental thyroid cancer or metastasis with regard to treatment options, risk of complications and adverse effects and prognosis. Patients who are more engaged in their health care decision making are more likely to experience confidence in treatment decisions, satisfaction with treatment, and trust in their providers (87). Our study showed a strong preselection of patients eligible for FNAC and surgery, indicating that further investigations were performed only if the results had impact on treatment algorithms. Similarly, two other studies demonstrated that ^18^F-FDG PET/CT incidental findings could be managed appropriately in the clinical context and based on physician and patient decisions (88, 89).

When aiming at allocating FTI for FNAC, ultrasound classification systems might be valuable. They have been developed in order to improve the uniformity of the interpretation of the sonographic patterns and the stratification of thyroid nodules for FNAC. These ultrasound-based tools have been validated in the general population of patients with nodular goiter and an estimated cancer prevalence of 2-3% (90). As shown in the present study, thyroid cancer prevalence among patients with FTI at ^18^F-FDG PET/CT is significantly higher. Since the pretest risk of malignancy is hence higher for the latter the aim of the classification system will change likewise from saving unnecessary FNAC to detecting malignancy accurately. Four included studies aimed to assess the reliability of ultrasound classification systems in indicating FNAC and predicting malignancy in FTI on ^18^F-FDG PET/CT (58, 71, 72, 83). Three of them demonstrated, that the malignancy risk of FTI detected on ^18^F-FDG PET/CT in the low suspicion categories did not show an increase in malignancy when compared with the estimated malignancy risks of these categories suggested by the guidelines (58, 71, 72). The FTI belonging to these categories accounted for 30-37% of the total. Conversely, in two of the studies FTI detected on ^18^F-FDG PET/CT with intermediate to high suspicion showed an increase in malignancy in comparison with the estimated malignancy risks suggested by the guidelines (58, 72). Furthermore, Trimboli et al. compared three ultrasound classifications in indicating FNAC in FTI and showed that all had a good performance, possibly reducing unnecessary FNACs in 25-53% of the total (83). Though subject to limitations with regard to study design these preliminary results show that the implementation of ultrasound classification systems might contribute to less unnecessary FNACs in the low suspicious nodules, whereas the indications for FNACs of the intermediate or high suspicious nodules might be more evidenced. Guidelines are concordant in recommending against routine FNAC of nodules smaller than 1 cm, even if they are highly suspicious on ultrasound (12–14, 91).

Regarding the ROIF ultrasound classification systems might stratify thyroid nodules with BI, BIII and BIV. Guidelines recommend a repeat FNAC after a non-diagnostic initial FNAC. However, repeat US might be considered as well when initial European Thyroid Association Guidelines for Ultrasound Malignancy Risk Stratification of Thyroid Nodules in Adults (EU-TIRADS) is 2 or 3 (92). In case of BIII and BIV clinical management is not that straightforward. Several studies evaluated the usefulness of the ultrasound classification systems in predicting malignancy of thyroid nodules with indeterminate cytology according to the Bethesda classification (93–98). The varying results between the studies are affected by differences in sonographic patterns, cytologic diagnose and ROM. Even so, the US classifications confirm more or less a gradation in the pretest risk of malignancy. Therefore, it might be possible to guide management after an indeterminate cytological diagnosis based on US patterns. In other words, an intermediate or high suspicious ultrasound in a nodule with indeterminate cytology should trigger repeat FNAC or surgery, whereas a nodule with benign appearance may need clinical follow-up. Guidelines have not recommended this sonographic pattern stratification of nodules with indeterminate cytology and decision making should be made from a multidisciplinary perspective (14).

A new technique to manage indeterminate nodules could be the use of molecular markers. For example, BRAF mutation analysis could guide towards accurate surgical therapy. These molecular tests require standardization of performance characteristics and appropriate calibration as well as analytic validation before clinical interpretation (18, 99). Therefore, the routine BRAF testing does not (yet) have a place in the clinical routine and is therefore not recommended (100).

Some considerations in the interpretation of the results of the present systematic review and meta-analysis should be mentioned. First, a threat to the validity of any meta-analysis is publication bias. Our analyses were not suggestive of publication bias.

Second, the prevalence of the included studies showed substantial heterogeneity. Only age was a significant discriminator with studies with a mean age younger than 60 years having a higher prevalence of FTI. This finding might seem surprising given the fact that the prevalence of thyroid nodules increases with age (101). However, at the same time the prevalence of malignant, and therefore FDG-avid, thyroid nodules decreases with age (102). The subgroups were not controlled for contributing factors, such as sex distribution, histopathology or cytopathology findings, clinical signs of thyroid malignancy or risk factors for developing thyroid cancer, hampering straightforward conclusions (103). Another contributing factor might have been the applied definition of focal increased uptake in the thyroid gland on ^18^F-FDG PET/CT. Most studies used visual and semiquantitative assessments, which might be prone to non-replicability and variability of results. Patient selection, ^18^F-FDG PET/CT indication and geographic influences were of minor significance at subgroup analysis.

Third, a major limitation in calculating the ROM was the high degree of preselection of FTI for cyto- or histopathology and the different reference standards for defining malignancy. Although FNAC is valuable by facilitating the diagnostic correlation with histopathology, cytopathology is not considered the gold standard (104–107). Nevertheless, in the present meta-analysis both cyto- and histopathology results were used equally for estimating the ROM. ROM was not calculated using only histopathology results, because most patients undergoing diagnostic surgery were preselected by FNAC. Follicular carcinomas, which are per definition not higher than Bethesda IV, were still included in analysis as Bethesda IV often led to diagnostic surgery.

Fourth, the ROM of the selected studies showed moderate heterogeneity. This might be caused by the retrospective design of most studies with higher risks of bias and non-replicability of methods and results. The visual assessment method for defining FTI on ^18^F-FDG PET/CT might also have contributed as the degree of focal uptake of FTI might be of predictive though not of conclusive value for malignancy (10, 34, 37, 41, 45, 49).

Finally, only one fourth of included studies were suitable for analysis of the ROIF. Pooling of data resulted in substantial heterogeneity. No sources of heterogeneity could be shown at subgroup analysis. Variability in ROIF might be accounted to the hospital setting (i.e. settings of local multidisciplinary guidelines and consultations and organization of patient flow pathways), the degree of experience of the radiologist performing the FNAC, the availability of a cytopathology technician for on-site assessment of the specimen adequacy and a pathologist for consulting a second-reading of the FNAC. The latter might be of decisive importance as intra- and interobserver variation exists in the distinction between BIII and BIV (108).

The present systematic review and meta-analysis shows that FTI are a common finding on ^18^F-FDG PET/CT. Nodules are malignant in approximately one third of cases with the majority being PTC. At the same time, cytology results are non-diagnostic or indeterminate in one fifth of FNACs. Before proceeding to active examination of the FTI, the clinical context and the preferences of the patient should be reviewed and balanced with the possible scenarios after FNAC and the clinical impact of diagnosing PTC.

## Data Availability Statement

The original contributions presented in the study are included in the article/**Supplementary Material**. Further inquiries can be directed to the corresponding author.

## Author Contributions

Conception or design of the work, JL and HW. Data collection, JL, MM, and HW. Data analysis and interpretation, JL, MM, AH, AB, SK, BH, TL, and HW. Drafting the article, JL, MM, and HW. Critical revision of the article, JL, MM, AH, AB, SK, TL, and HW. Final approval of the version to be published, JL, MM, AH, AB, SK, BH, TL, and HW. All authors contributed to the article and approved the submitted version.

## Funding

The open access publication fee is funded by the Medical Imaging Center (MIC), which is a UMCG research facility. No other sources of funding were used.

## Conflict of Interest

The authors declare that the research was conducted in the absence of any commercial or financial relationships that could be construed as a potential conflict of interest.

## Publisher’s Note

All claims expressed in this article are solely those of the authors and do not necessarily represent those of their affiliated organizations, or those of the publisher, the editors and the reviewers. Any product that may be evaluated in this article, or claim that may be made by its manufacturer, is not guaranteed or endorsed by the publisher.
