# Supplementary material for: Focal Thyroid Incidentalomas on 18F-FDG PET/CT: A Systematic Review and Meta-Analysis on Prevalence, Risk of Malignancy and Inconclusive Fine Needle Aspiration
Source: Front Endocrinol (Lausanne). 2021 Oct 20;12:723394. doi: 10.3389/fendo.2021.723394 (PMC8564374; doi:10.3389/fendo.2021.723394)
Supplement: Supplementary file 1 [file Table_1.docx]

**Supplemental table 1** – Search strategies

| **Database** | **Search strategy** |
| --- | --- |
| MEDLINE/PubMed | ("Thyroid Neoplasms"[Mesh] OR "Thyroid Gland"[Mesh] OR thyroid[tiab])AND ("Positron-Emission Tomography"[Mesh] OR "Fluorodeoxyglucose F18"[Mesh] OR positron emission tomograph*[tiab] OR pet[tiab] OR 18F-FDG[tiab] OR 18-F-FDG[tiab] OR 18F-fluorodeoxyglucose[tiab] OR 18-F-fluorodeoxyglucose[tiab]) AND ("Incidental Findings"[Mesh] OR incident*[tiab]) |
| Embase | ('thyroid tumor'/exp OR 'thyroid gland'/exp OR thyroid:ti,ab)  AND  ('positron emission tomography'/exp OR 'PET-CT scanner'/exp OR 'fluorodeoxyglucose f 18'/exp OR "positron emission tomograph*":ti,ab OR pet:ti,ab OR ‘18F-FDG’:ti,ab OR ‘18-F-FDG’:ti,ab OR ‘18F-fluorodeoxyglucose’:ti,ab OR ‘18-F-fluorodeoxyglucose’:ti,ab)  AND  ('thyroid incidentaloma'/exp OR 'incidental finding'/exp OR incident*:ti,ab)NOT 'conference abstract'/it |
| Web of Science | TS=("thyroid")  AND  TS=("Positron-Emission Tomography" OR "Fluorodeoxyglucose F18" OR "positron emission tomograph*" OR "pet" OR "18F-FDG" OR "18-F-FDG" OR "18F-fluorodeoxyglucose" OR "18-F-fluorodeoxyglucose")  AND  TS=("Incident*")  NOT  DT=("meeting abstract") |
