# Supplementary material for: Focal Thyroid Incidentalomas on 18F-FDG PET/CT: A Systematic Review and Meta-Analysis on Prevalence, Risk of Malignancy and Inconclusive Fine Needle Aspiration
Source: Front Endocrinol (Lausanne). 2021 Oct 20;12:723394. doi: 10.3389/fendo.2021.723394 (PMC8564374; doi:10.3389/fendo.2021.723394)
Supplement: Supplementary file 2 [file Table_2.docx]

**Supplemental table 2** – QUADAS-2 assessment

|  | **Risk of Bias** | | | |  | **Applicability** | | |
| --- | --- | --- | --- | --- | --- | --- | --- | --- |
|  | **Patient Selection** | **Index Test** | **Reference Test** | **Flow and Timing** |  | **Patient Selection** | **Index Test** | **Reference Test** |
| Kim et al., 2010 | Low | Low | High | Low |  | Low | Low | Low |
| Kung et al., 2010 | High | Unclear | High | High |  | Low | Low | Low |
| Zhai et al., 2010 | Low | Low | High | High |  | Low | Low | Low |
| Czepczyński et al., 2011 | Low | Low | High | High |  | Low | Low | Low |
| Hsiao et al., 2011 | High | Low | High | High |  | Low | Low | Low |
| Kim et al., 2011 | High | Low | High | Low |  | Low | Low | Low |
| Nilsson et al., 2011 | High | Low | High | Low |  | Low | Low | Low |
| Nishimori et al., 2011 | Low | Unclear | Low | High |  | Low | Low | Low |
| Pagano et al., 2011 | High | Low | High | High |  | Low | Low | Low |
| Prichard et al., 2011 | Low | Unclear | High | Low |  | Low | Low | Low |
| Wong et al., 2011 | Low | Low | High | High |  | Low | Low | Low |
| Boeckmann et al., 2012 | Low | Low | High | High |  | Low | Low | Low |
| Bonabi et al., 2012 | Low | Unclear | High | Low |  | Low | Low | Low |
| Fujii et al., 2012 | Low | Low | High | Low |  | Low | Low | Low |
| Kao et al., 2012 | Low | Low | High | High |  | Low | Low | Low |
| Lee et al., 2012 | High | Low | Low | Low |  | Low | Low | Low |
| Pampaloni et al., 2012 | High | Unclear | High | High |  | Low | Low | Low |
| Yang et al., 2012 | Low | Low | High | High |  | Low | Low | Low |
| Bertagna et al., 2013 | Low | Low | High | High |  | Low | Low | Low |
| Kim et al., 2013 | High | Low | Low | Low |  | Low | Low | Low |
| Achury et al., 2014 | Low | Low | High | High |  | Low | Low | Low |
| Brindle et al., 2014 | Low | Unclear | High | High |  | Low | Low | Low |
| Choi et al., 2014 | High | Low | Low | Low |  | Low | Low | Low |
| Elzein et al., 2015 | Low | Unclear | High | High |  | Low | Low | Low |
| Marques et al., 2014 | Low | Low | High | High |  | Low | Low | Low |
| Stangierski et al., 2014 | Low | Low | Low | Low |  | Low | Low | Low |
| Yaylali et al., 2014 | Low | Low | High | High |  | Low | Low | Low |
| Adas et al., 2015 | Low | Unclear | High | High |  | Low | Low | Low |
| Agrawal et al., 2015 | Low | Unclear | High | High |  | Low | Low | Low |
| Chun et al., 2015 | Low | Low | High | Low |  | Low | Low | Low |
| Gavriel et al., 2015 | High | Low | High | Low |  | Low | Low | Low |
| Jamsek et al., 2015 | Low | Low | Low | High |  | Low | Low | Low |
| Kim et al., 2015 | Low | Low | Low | Low |  | Low | Low | Low |
| Kim et al., 2015 | High | Low | Low | High |  | Low | Low | Low |
| Sharma et al., 2015 | Low | Low | High | Low |  | Low | Low | Low |
| Yoon et al., 2015 | High | Low | Low | Low |  | Low | Low | Low |
| Barrio et al., 2016 | High | Low | High | High |  | Low | Low | Low |
| Demir et al., 2016 | Low | Low | High | Low |  | Low | Low | Low |
| Flukes et al., 2016 | Low | Unclear | Low | High |  | Low | Low | Low |
| Hassan et al., 2016 | High | Low | Low | High |  | Low | Low | Low |
| Şencan Eren et al., 2016 | High | Low | Low | High |  | Low | Low | Low |
| Vaish et al., 2016 | Low | Low | Low | High |  | Low | Low | Low |
| Hagenimana et al., 2017 | Low | Unclear | Low | Low |  | Low | Low | Low |
| Li et al., 2017 | High | Unclear | Low | High |  | Low | Low | Low |
| Makis et al., 2017 | High | Low | High | High |  | Low | Low | Low |
| Ozderya et al., 2017 | Low | Low | Low | Low |  | Low | Low | Low |
| Pak et al., 2017 | High | Low | Low | Low |  | Low | Low | Low |
| Sollini et al., 2017 | High | Low | High | High |  | Low | Low | Low |
| Thuillier et al., 2017 | Low | Low | Low | High |  | Low | Low | Low |
| Chung et al., 2018 | High | Low | Low | High |  | Low | Low | Low |
| Pattison et al., 2018 | Low | Unclear | Low | High |  | Low | Low | Low |
| Sager et al., 2018 | Low | Unclear | High | Low |  | Low | Low | Low |
| AbdelLowHalim et al., 2019 | Low | Unclear | Low | Low |  | Low | Low | Low |
| Kumar et al., 2019 | High | Low | Low | Low |  | Low | Low | Low |
| Larg et al., 2019 | Low | Low | Low | High |  | Low | Low | Low |
| Lin et al., 2019 | Low | Low | Low | Low |  | Low | Low | Low |
| Oven et al., 2019 | High | Unclear | High | Low |  | Low | Low | Low |
| Shi et al., 2019 | High | Low | High | Low |  | Low | Low | Low |
| Bakhshayesh Karam et al., 2020 | Low | Low | High | High |  | Low | Low | Low |
| Kamakshi et al., 2020 | Low | Low | Low | High |  | Low | Low | Low |
| Trimboli et al., 2020 | High | Low | High | Low |  | Low | Low | Low |
